# Improvement in NO_2_ Gas Sensing Properties of Semiconductor-Type Sensors by Loading Pt into BiVO_4_ Nanocomposites at Room Temperature

**DOI:** 10.3390/ma14205913

**Published:** 2021-10-09

**Authors:** Wang-De Lin, Shu-Yun Lin, Murthy Chavali

**Affiliations:** 1 Department of Center for General Education, St. Mary’s Junior College of Medicine, Nursing and Management, Yilan City 26647, Taiwan; 2 Department of Applied Chemistry, Providence University, Taichung City 43301, Taiwan; s1063051@gm.pu.edu.tw; 3 Office of the Dean (Research) & Division of Chemistry, Department of Sciences, Faculty of Sciences & Technology, Alliance University, Karnataka, Bengaluru 562106, India; siva.chavali@alliance.edu.in or; 4 NTRC-MCETRC and 109 Composite Technologies Pvt. Ltd., Andhra Pradesh, Guntur District, Guntur 522201, India

**Keywords:** NO_2_, gas sensors, Pt/BiVO_4_, nanocomposite

## Abstract

In the present study, we report the first attempt to prepare a conducive environment for Pt/BiVO_4_ nanocomposite material reusability for the promotion of sustainable development. Here, the Pt/BiVO_4_ nanocomposite was prepared using a hydrothermal method with various weight percentages of platinum for use in NO_2_ gas sensors. The surface morphologies and structure of the Pt/BiVO_4_ nanocomposite were characterized by scanning electron microscope (SEM), transmission electron microscopy (TEM), energy-dispersive X-ray spectroscopy (EDX), and X-ray diffraction (XRD). The results showed that Pt added to BiVO_4_ with 3 wt.% Pt/BiVO_4_ was best at a concentration of 100 ppm NO_2_, with a response at 167.7, and a response/recovery time of 12/35 s, respectively. The Pt/BiVO_4_ nanocomposite-based gas sensor exhibits promising nitrogen dioxide gas-sensing characteristics, such as fast response, highly selective detection, and extremely short response/recovery time. Additionally, the mechanisms of gas sensing in Pt/BiVO_4_ nanocomposites were explored in this paper.

## 1. Introduction

Nitrogen dioxide (NO_2_) emissions are largely the result of fossil fuel combustion in automobiles and electrical power plants [1,2]. NO_2_ exposure at concentrations as low as a few ppm can be dangerous to human health and higher concentrations have been linked to smog and acid rain [3,4]. In the current study, we developed a highly sensitive NO_2_ sensor capable of detection at extremely low concentrations at room temperature [5,6,7]. One common approach to NO_2_ detection involves nanostructures of bismuth vanadate (BiVO_4_). Beyond NO_2_ detection [8,9,10], BiVO_4_ has been used in a wide variety of applications, such as formaldehyde degradation [11,12], H_2_S production [13,14], hydrogen production [15,16], CO_2_ capture [17,18], and other fields [19,20,21]. Table 1 lists a variety of NO_2_ sensors based on Pt or BiVO_4_. BiVO_4_ is inexpensive, highly responsive to visible light, stable, non-toxic, and environmentally benign with a narrow bandgap of Eg = 2.4 eV [22]. However, the low charge transfer rate of BiVO_4_ impedes efforts to enhance photocatalytic activity [23].

To improve charge transfer, various methods have been planned, including doping with various metals [24,25], the surface deposition of noble metals [26,27,28], and the formation of compound semiconductors [29,30]. The use of dopants has been shown to introduce electronic barriers, which capture photogenerated electrons and transfer them to other materials to prevent electron-hole recombination.

In this study, we developed a novel Pt/BiVO_4_ composite designed specifically for the sensing of NO_2_ at room temperature.

## 2. Materials and Methods

### 2.1. Materials

(Bi(NO_3_)_3_·5H_2_O), (NH_4_VO_3_), ethanol (C_2_H_5_OH), nitric acid (HNO_3_) ammonium hydroxide (NH_4_OH), sodium borohydride (NaBH_4_), ethylenediaminetetraacetic acid (EDTA), silver(I) nitrate (AgNO_3_) and dihydrogen hexachloroplatinate (VI) hexahydrate (H_2_PtCl_6_·6H_2_O) were purchased from Sigma-Aldrich Co., Inc. (St. Louis, MO, USA). Distilled H_2_O obtained using the distillation water system provided by the Millipore Corporation (Millipore Corp. Molsheim, France) was used.

### 2.2. Preparation of Bismuth Vanadate

Briefly, a few mmol of Bismuth nitrate (4 g) was added to 6.8 mmol of EDTA (2 g) in acidic medium (0.3 M), whereupon gradual heating to 85 °C was performed for the mixture to procure a colourless solution (named Solution A). A total of 8.5 mmol (1 g) of NH_4_VO_3_ was then dissolved in 50 mL of H_2_O at 60 °C under vigorous stirring for the generation of a yellow solution (named Solution B). The above two solutions mentioned (i.e., Solution A + Solution B) were then further processed with mixing at 50 °C for 1 hour with controlled stirring, after which, the resulting mixture was controlled with pH adjustment to 3.0 by the addition of salt (1 M). The prepared mixed product was poured into the autoclave (Dogger, New Taipei City, Taiwan) made of stainless steel with Teflon lining and further covered with sealant, then kept for heating, which was maintained at 180 °C for 6 h. After cooling of the autoclave, the resultant product was withdrawn via centrifugation, before undergoing multiple washings using ultrapure water and ethanol, followed by drying in an oven at 80 °C overnight and calcination at 450 °C for 4 h to complete the synthesis of BiVO_4_ [11].

### 2.3. Synthesis Method for Pt/BiVO_4_ Nanocomposite

Distilled water containing a pre-calculated quantity of H_2_PtCl_6_⋅6H_2_O was added to the as-prepared product of BiVO_4_, which was dispersed in 100 mL and further maintained for 1 h at a continuous stirring rate. The precursor suspension was produced as the product was extracted for centrifugation, washed three times using DI water, and then three times again using alcohol. The precipitate obtained was prepared by drying at 80 °C for more than 6 h. Pt/BiVO_4_ preparation was done with Pt at various weight concentrations labelled as follows: 0, 0.5, 1, 3, 5, or 10 wt.% Pt/BiVO_4_.

### 2.4. Characterization

The proposed Pt/BiVO_4_ nanocomposite was determined using a transmission electron microscope for morphology and structure with an energy-dispersive X-ray spectroscope (TEM/EDS; JEM-2100F), and a field emission scanning electron microscope (FESEM; JEOL JSM-7500F) operated at 30 kV. Pt/BiVO_4_ was characterized to understand its crystal structure using a Shimadzu X-ray diffractometer at 1.5405 Å at 40 kV and 30 mA, supported by a vertical goniometer in the range of 10° to 80° (2theta) at a scan speed of 2 °/min.

### 2.5. Sensor Fabrication and Measurements

The designed sensors were prepared by a dipping and coating procedure (Binder: PVA) on an alumina-based solid substrate (10 × 5 mm^2^; and fab of chip-based sensor; rotational speed, 1000 rpm) in the prepared material, to fabricate an electrode with a comb-like structure. The chips were subsequently pretreated to 80 °C over a period of 0.5 h and then calcined at a temperature of 400 °C for 2 h.

**Figure 1:**
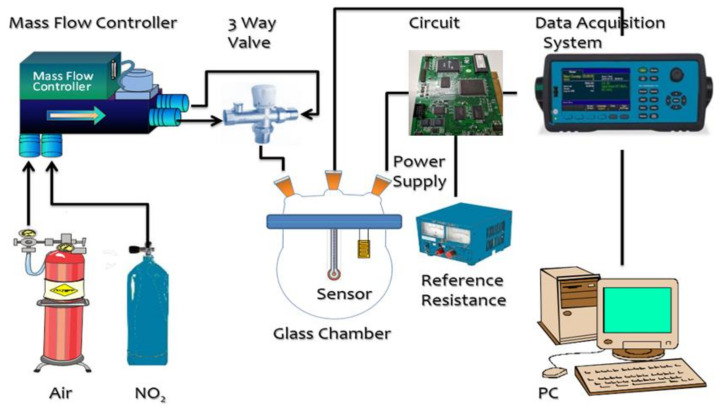
Diagrammatic representation of the experimental setup.

Figure 1 presents a schematic diagram showing the experimental setup used to measure the electrical response of the sensors [3]. A homemade arrangement for the sensor in the glass chamber was designed with a dynamic flow rate system to evaluate gas sensing performance. The target gas was injected into the chamber at the desired concentrations with a design including a mass controller arrangement for controlling the flow rate (1, 10, 30, 50, 70, or 100 ppm NO_2_) via a hole in the cover of the chamber. A simple circuit was utilized to understand signals coming from the head of the sensor, which is further showcased by resistance values; later on, collection of data for evaluation and processing was done with a PC. All resistance measurements were averaged from multiple measurements obtained using a Jiehan5000 data acquisition system with the input voltage from a power supply (Vs) set at 4.0 V. S = Rg/Ra—which is the ratio that is used for the calculation of the response from the sensor [31], where Ra indicates the resistance in the presence of air and Rg indicates the resistance in the presence of NO_2_ gas in the provided system. The times required for a 90% variation in resistance upon exposure to NO_2_ or air are described as the response and recovery times. Selectivity toward NO_2_ was assessed by exposing the sensor individually, including carbon monoxide, nitric oxide, methane (concentrations equal to 100 ppm), and further recording the characteristics of the response with corresponding values. Long-term stability was assessed by repeating the sensing measurements on multiple consecutive days.

## 3. Results and Discussion

### 3.1. Structure Property

**Figure 2:**
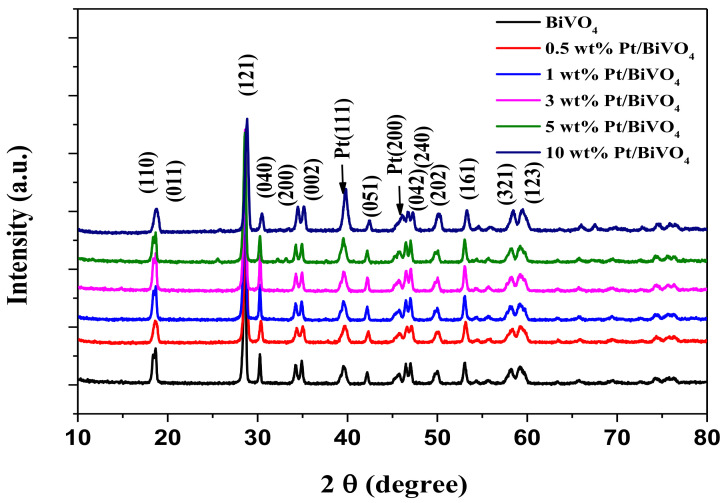
XRD patterns of different contents for Pt/BiVO_4_ nanocomposites.

Figure 2 shows XRD measurements, indicating the phases and structure of pure BivO_4_ and various Pt/BiVO_4_ nanocomposites. The samples exhibited characteristic peaks corresponding to a structure of BiVO_4_ (JCPDS 14-0688) with a monoclinic arrangement, as follows: 18.6° (110), 19.0° (011), 29.3° (121), 30.5° (040), 34.5° (200), 35.2° (002), 39.5° (211), 43.2° (051), 46.0° (042), 47.6° (240), 50.3° (202), 53.6° (161), 57.9° (321), and 59.3° (132) [19]. Peaks at 39.8° (111) and 46.0° (200) correspond to face-centred cubic Pt (JCPDS card: No. 04-0802) [32]. No other impurity peak was detected, indicating that the prepared samples were of high purity. Moreover, with increases in the amounts of deposited Pt, the diffraction peaks of Pt (111) were gradually intensified. Using Scherrer’s equation, the mean Pt (111), BiVO_4_ (051) and (161) crystalline sizes were estimated and listed in Table 1. Table 1 reveals that the crystalline sizes of Pt wt.% loading at 0.5%, 1%, 3%, 5% and 10% were 10.1 nm, 10.6 nm, 12.1 nm, 12.9 nm and 16.3 nm, respectively. The BiVO_4_ (051) and (161) crystalline sizes were 24.2–25.8 nm and 25.8–28.2 nm, respectively. This shows that the Pt (111) crystalline size was increased by enlarging the amount of Pt loading. However, the BiVO_4_ crystal size did not change greatly with the addition of platinum. These results indicate that the sensing material was a simple mixture.

**Figure 3:**
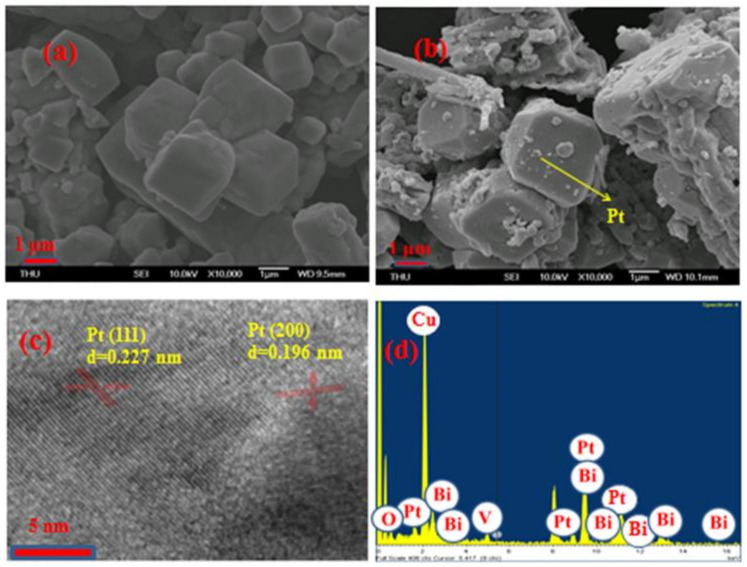
(**a**) FESEM images of BiVO_4_ (**b**) Pt/BiVO_4_; (**c**) TEM images of Pt/BiVO_4_ (**d**) EDS spectrum of 3wt.% Pt/BiVO_4_ nanocomposites.

The morphology and microstructure of the BiVO_4_ and Pt/BiVO_4_ were characterized by FESEM and TEM evaluation of the nanocomposite structures. As shown in Figure 3a, SEM images revealed an arrangement comprising a large number of irregularly stacked BiVO_4_ structures with an average diameter of 0.7–2.5 μm. Figure 3b shows the Pt/BiVO_4_ nanocomposite containing Pt particles, with uneven diameters on the BiVO_4_ sample. As shown in Figure 3c, TEM micrographs revealed d-spacing in the prescribed range of 0.227 and 0.196 nm, respectively corresponding to the original Pt-based (111) and (200) lattice plane [32]. As shown in Figure 3d, the EDS spectra confirmed the presence of Pt, V, Bi, Cu and O. The presence of strong signals from Cu can be ascribed to the Cu grid. Taken together, these confirm the formation of fully developed nanocomposites with a pure phase structure.

### 3.2. Gas-Sensing Performance of Pt/BiVO_4_

**Figure 4:**
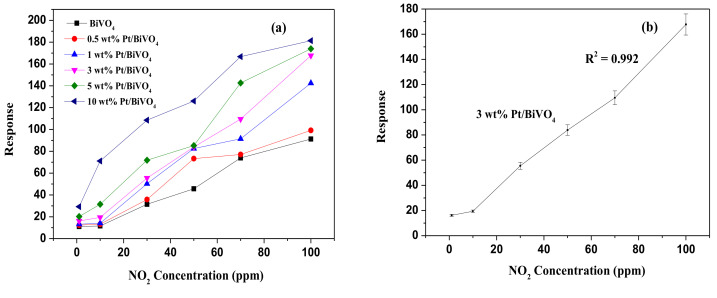
(**a**) Discrepancies in response relative to variations in 1–100 ppm NO_2_ for varying Pt/BiVO_4_ nanocomposites contents. (**b**) Response of the 3wt.% Pt/BiVO_4_ sensor to 1–100 ppm NO_2_ at room temperature.

Figure 4a illustrates fluctuations in the response of sensors comprising (0, 0.5, 1, 3, 5 and 10) wt.% Pt/BiVO_4_ nanocomposites following exposure to NO_2_ gas at concentrations of 1–100 ppm at 25 °C. The increased response at all gas concentrations is indicative of typical n-type semiconductor behavior. The unloaded BiVO_4_ materials exhibited a strong response (91.3) when exposed to high concentrations of NO_2_ gas (100 ppm). Loading samples with a high concentration of Pt (10 wt.% Pt) increased the response strength substantially to 181.4. Table 1 lists the response times, recovery times, sensor responses, and base linearity results. The response and recovery times of sensors based on Pt/BiVO_4_ nanocomposites were shorter than those of Pt/BiVO_4_ nanocomposites. The 3 wt.% Pt/BiVO_4_ nanocomposites achieved a T_90_ of only 12 s and a Tr_90_ of 35 s. As shown in Figure 4b, this sample also presented the highest linearity (R^2^ = 0.992) and sensor response (S = 167.7). 

Note that the response and recovery times of samples with high Pt concentrations (5 wt.% and 10 wt.%) were slower than those of the 3 wt.% Pt/BiVO_4_ nanocomposite. These results are similar to those reported in [31,33], indicating that at higher Pt concentrations, the formation of Pt aggregates tends to hinder the response and recovery of Pt/BiVO_4_ nanocomposites. Overall, the 3 wt.% Pt/BiVO_4_ sensor presented the best NO_2_ sensing performance and was therefore selected as the gas sensor material for all subsequent experiments.

**Figure 5:**
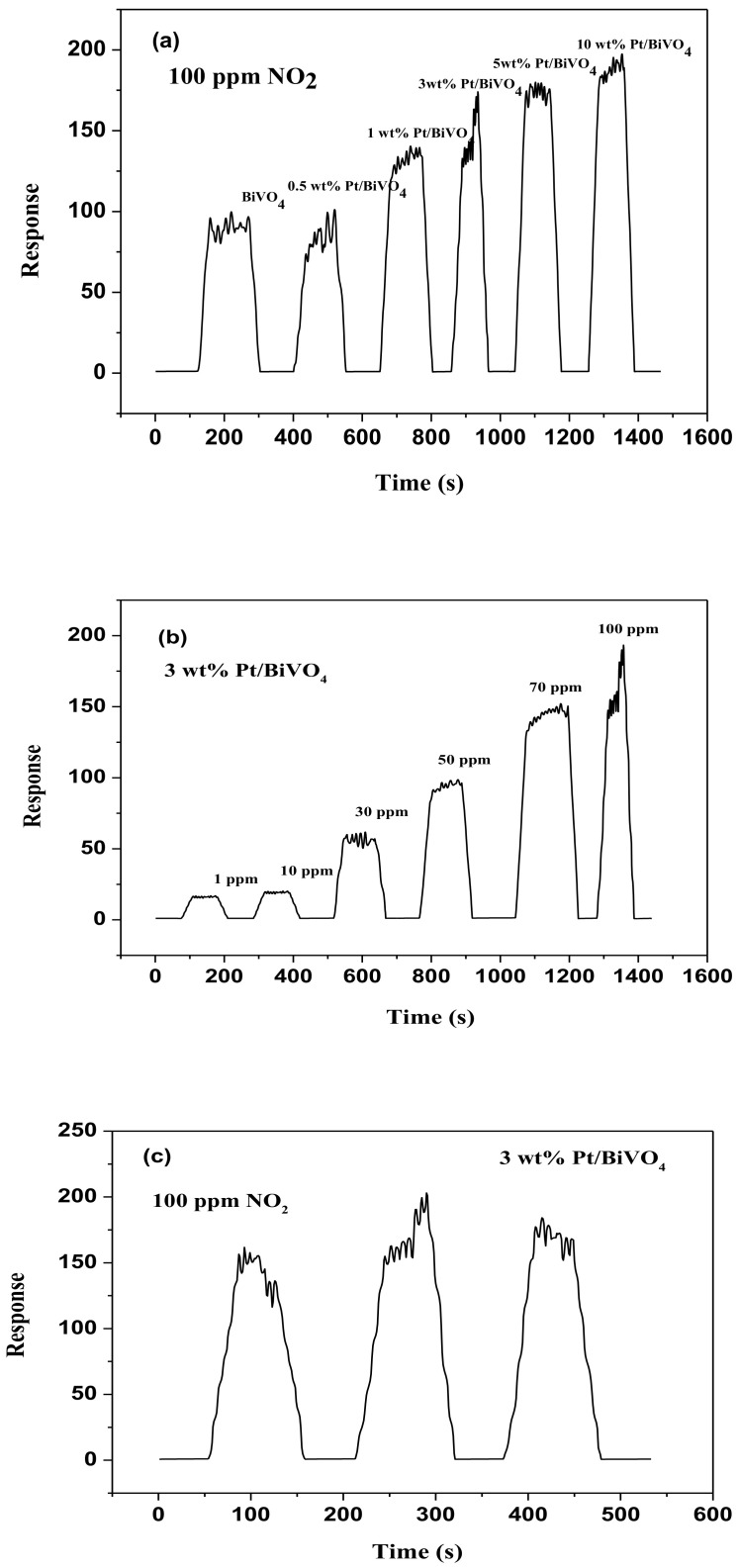
(**a**) The response for varying Pt/BiVO_4_ nanocomposites contents of 100 ppm nitrogen dioxide at room temperature; (**b**) Curves for responses of the 3wt.% Pt/BiVO_4_ in the range of 1–100 ppm nitrogen dioxide at room temperature; (**c**) Three-cycle repeated response curves of the 3wt.% Pt/BiVO_4_ to 100 ppm nitrogen dioxide at room temperature.

Response and recovery times are important parameters in sensor characterization and should be as short as possible. Figure 5a presents typical dynamic response curves as a function of the weight ratios of Pt/BiVO_4_ when exposed to NO_2_ at a concentration of 100 ppm at room temperature. Figure 5b presents the dynamic sensing curve of the Pt/BiVO_4_ sensor when exposed to NO_2_ gas at various concentrations (1–100 ppm). Table 1 lists sensing properties, sensor response times, recovery times, and linearity (R^2^). The dramatic response of the sensors upon exposure to NO_2_ is typical of n-type semiconductors.

We can see in Figure 5a that the strength of the responses increased with low NO_2_ concentrations (1–100 ppm), as shown in Figure 5b. As shown in Figure 5c, the results from the proposed sensor maintained high reproducibility throughout the test cycle, thereby demonstrating that the Pt/BiVO_4_ sensor could reliably be at this particular concentration level to monitor the concentration of NO_2_.

**Figure 6:**
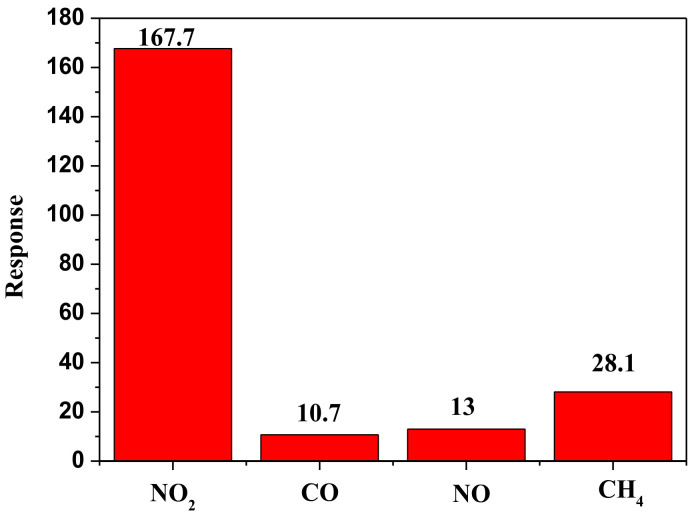
Selectivity test of the 3wt.% Pt/BiVO_4_ nanocomposites to 100 ppm of various gases at room temperature.

Gas sensors are utilized and mostly evaluated with a range of selectivity, which is an important parameter. Figure 6 illustrates the responses of 3 wt.% Pt/BiVO_4_ nanocomposites to NO_2_, CO, NO, and CH_4_ (100 ppm) at room temperature. Under these conditions, the response to NO_2_ was 167.7, whereas the responses provided by the other selected gases were all lower, at a value of 30—thereby confirming the extraordinary selectivity demonstrated in 3 wt.% Pt/BiVO_4_ nanocomposites to NO_2_.

**Figure 7:**
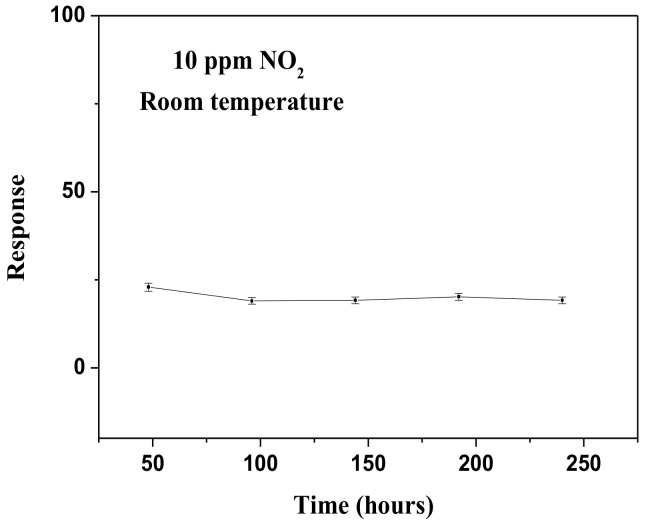
Stability of 3 wt.% Pt/BiVO_4_ nanocomposites to 10 ppm NO_2_ operating at room temperature.

As shown in Figure 7, we did not observe a significant decrease in the response of the 3 wt.% Pt/BiVO_4_ nanocomposite despite continuous exposure to NO_2_ (10 ppm) for a period of 10 days, thereby demonstrating the stability of the sensors.

### 3.3. Underlying Sensing Mechanism

This study demonstrated that the addition of Pb to BiVO_4_ notably enhanced the response characteristics of the sensors. We assume that these effects are described by the provided reaction mechanism (1)–(4) [9,26,31,34,35]:

(1) $$O_{2}\left(gas\right)+e^{-}\to O_{2}^{-}\left(ads\right)$$

(2) $$NO_{2}\left(gas\right)\to NO_{2}\left(ads\right)$$

(3) $$NO_{2}\left(ads\right)+O^{-}\left(ads\right)\to NO\left(gas\right)+O_{2}^{-}\left(ads\right)$$

(4) $$NO_{2}\left(gas\right)+O_{2}^{-}\left(ads\right)+2e^{-}\to NO_{2}^{-}\left(gas\right)+2O^{-}\left(ads\right)$$

**Figure 8:**
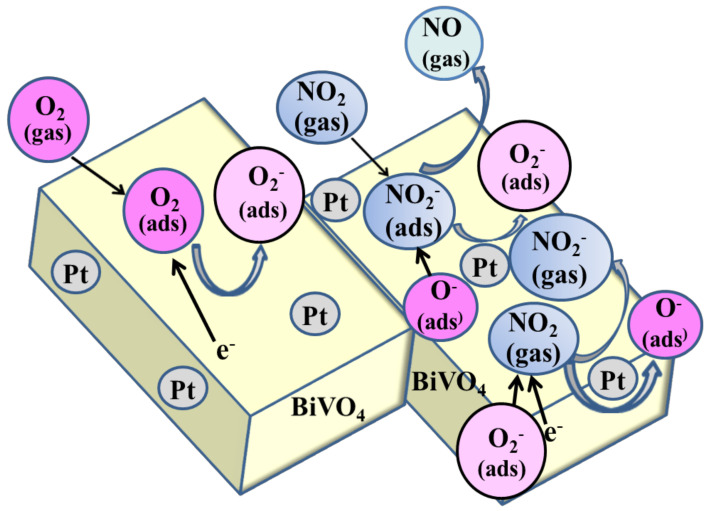
Schematic diagram of NO_2_ gas sensing mechanism for Pt/BiVO_4_ nanocomposite.

The enhancement mechanism was projected and illustrated in Figure 8. The Pt-based BiVO_4_ nanocomposite exhibited typical n-type semiconductor behaviour [9,36]. When exposed to air, the active sites on the BiVO_4_ started adsorbing the oxygen molecules, which in turn gave free electrons from the BiVO_4_ material for the process of chemisorbed behaviour of oxygen ions ($O_{2}^{-}$) at RT (Equation (1)). The subsequent adsorption of NO_2_ molecules at available adsorption sites on the BVO_4_ surface enables the direct extraction of the surface electrons of the prepared sensor, producing a breaking of bond in the form of NO—the result of which leaves free oxygen ions ($O_{2}^{-}$ *)* (Equations (2) and (3)) [37]. Catalysis of platinum can help in the enhancement of responsiveness of materials used for sensors to the various provided gases, due to a phenomenon referred to as the spillover effect [38,39]. The activity of Pt in this catalysis can help in accelerating the adsorption behaviour of molecular level oxygen; the adding of Pt dopants has a significant effect on surface area, which can lead to further chemisorbed oxygen species spillover, which increases the number of active sites on the BiVO_4_ [11,26,33,40]. This increases the number of electrons that are captured, and in so doing enhances the response of the sensor. As shown in Equation (4), the availability of additional oxygen molecules for reactions with incoming NO_2_ gas molecules increases the number of interactions between gas molecules in nitrous oxide and the sensing layer. The surface modification of BiVO_4_ with platinum also increases the number of dissociated NO_2_ molecules migrating to the surface of BiVO_4_, thereby increasing responsivity to NO_2_ gas. For comparison, the various NO_2_ sensors based on Pt or BiVO_4_ are listed in Table 2.

## 4. Conclusions

In this study, a Pt/BiVO_4_ nanocomposite was prepared using a hydrothermal method with various weight percentages of platinum for use in NO_2_ gas sensors. The structure and morphology of samples were characterized by XRD, TEM, FESEM, and EDX. The experiment results demonstrated that the addition of Pt to BiVO_4_ at a concentration of 3 wt.% can greatly enhance the responsivity of Pt/BiVO_4_ nanocomposite sensors to NO_2_ at relatively low concentrations (100 ppm) as follows: sensor response (167.7), response time (12 s), and recovery time (35 s). The proposed Pt/BiVO_4_ nanocomposite-based gas sensor exhibited promising nitrogen dioxide gas-sensing characteristics, including high sensitivity, high selectivity, and extremely short response/recovery times.

## Figures and Tables

**Table 1:** Comparison of the NO_2_-sensing performance of different contents of Pt/BiVO_4_ nanocomposites.

Sensing Material	Response Time (T_90_, s)	Recovery Time (T_r90_, s)	Response (100 ppm)	Pt Crystalline Size (by XRD) (nm), hkl, 111	Bi Crystalline Size BiVO_4_ (by XRD) (nm), hkl		Linearity (R^2^)
					(051)	(161)	
BiVO_4_ 0.5 wt.% Pt/BiVO_4_ 1 wt.% Pt/BiVO_4_ 3 wt.% Pt/BiVO_4_ 5 wt.% Pt/BiVO_4_ 10 wt.% Pt/BiVO_4_	72 79 64 12 95 43	44 24 51 35 102 67	91.3 99.2 143.5 167.7 173.9 181.4	- 10.1 10.6 12.1 12.9 16.3	24.8 24.2 25.2 25.5 25.8 24.6	26.5 25.8 27.7 27.7 28.2 27.4	0.983 0.950 0.982 0.992 0.980 0.928

**Table 2:** Comparison of working temperature and various NO_2_ sensors based on Pt or BiVO_4_ nanocomposites.

Sensing Material	Response (Rg/Ra or Ra/Rg)	NO_2_ (ppm)	Temperature (°C)	References
Pt-SnO_2_ α-Fe_2_O_3_/BiVO_4_ BiVO_4_/Cu_2_O BiVO_4_/Cu_2_O/rGO rGO-NiO-BiVO_4_ Pt/WO_3_	1.3 7.8 4.2 8.1 8.1 11.24	30 2 4 1 2 1	50 110 60 60 60 150	[41] [10] [34] [9] [7] [35]
Pt/BiVO_4_	167.7	100	25	This work

## Data Availability

The data presented in this study are available on request from the corresponding author. The data are not publicly available due to a complicated structure that requires additional explanations.
